# Receptor-like cytoplasmic kinases mediated signaling in plant immunity: convergence and divergence

**DOI:** 10.1007/s44154-025-00219-8

**Published:** 2025-06-16

**Authors:** Juan Wang, Lu Bai, Yuchen Xu, Xinhang Zheng, Wenfeng Shan, Xuetao Shi, Shoucai Ma, Jiangbo Fan

**Affiliations:** 1 Shanghai Collaborative Innovation Center of Agri-Seeds, School of Agriculture and Biology, Shanghai Jiao Tong University, Shanghai, China; 2 Xianghu Laboratory, Hangzhou, China

**Keywords:** Receptor-like cytoplasmic kinase, Pattern-triggered immunity, Effector-triggered immunity, Signal convergence, Signal divergence, Plant immunity

## Abstract

Receptor-like cytoplasmic kinases (RLCKs) function as a central player in plant receptor kinases-mediated signaling, which regulate various aspects of plant immunity and growth. RLCKs receive signals from pattern recognition receptors (PRRs) to activate pattern-triggered immunity (PTI), including reactive oxygen species (ROS) production, Ca^2+^ influx, mitogen-activated protein kinase (MAPK) cascades, cellulose synthesis, phosphatidic acid (PA) production, hormone synthesis and signaling, and transcriptional remodeling. Besides, RLCK also participate in effector-triggered immunity (ETI) and the interplay between ETI and PTI. Increasing evidences show that much more RLCKs are involved in plant immune responses and form an intertwined signaling network. This review summarizes the recent findings about RLCKs-mediated signaling in plant immune responses and emphasizes signal convergence and divergence involved which provides new insights into the RLCKs signaling network in diverse biological processes.

**Supplementary Information:**

The online version contains supplementary material available at 10.1007/s44154-025-00219-8.

## Introduction

Plant immune responses help defend against pathogen infections by pattern-triggered immunity (PTI) and effector-triggered immunity (ETI). PTI and ETI are initiated by cell surface-localized PRRs and intracellular nucleotide-binding domain leucine-rich repeat containing receptors (NLRs) respectively (Yu et al. 2017; Yuan et al. 2021). Upon infection, PRRs recognize pathogen- or microbe- or damage-associated molecular patterns (PAMPs/MAMPs/DAMPs) to trigger host immune responses (PTI), including ROS production or ROS burst, Ca^2+^ influx, MAPK cascades, PA production, cell wall reinforcement, transcriptional remodeling, and others (Yu et al. 2017). While NLRs recognize pathogen effectors to trigger a more specific and robust immune response (ETI), usually leading to cell death, also known as the hypersensitive response (HR). ETI demonstrates stronger and more prolonged defense responses compared to PTI (Hatsugai et al. 2017). PTI and ETI constitute the two layers of plant immune system, and potentiate each other to enable hosts to establish a robust defense system against pathogen attacks (Chang et al. 2022; Yuan et al. 2021).

Receptor-like kinases (RLKs) and receptor-like proteins (RLPs) are the most common PRRs in terrestrial plants. RLKs and RLPs could recognize various ligands to regulate plant immune responses. The former contains an ectodomain (ECD) for signal perception, a transmembrane domain, and a cytoplasmic kinase domain for signal transduction, while the latter lacks an intracellular kinase domain (Dievart et al. 2020; He et al. 2018). RLCKs constitute a superfamily with RLK and RLP. Different from RLK and RLP, RLCKs are characterized by the absence of ECD. Although most RLCKs also lack the transmembrane domain, a few RLCK members do possess a transmembrane domain which allows membrane localization (Shiu and Bleecker 2001). RLCKs are potentially anchored to the plasma membrane through myristoylation or palmitoylation modification (Veronese et al. 2006). In total, 149 RLCK members are encoded in Arabidopsis genome and 379 in the rice genome which fall into 17 subfamilies according to sequence homology. Most RLCKs contain only one Ser/Thr kinase domain (Vij et al. 2008; Shiu et al. 2004).

## RLCKs function in PRR-mediated signaling

**Fig. 1:**
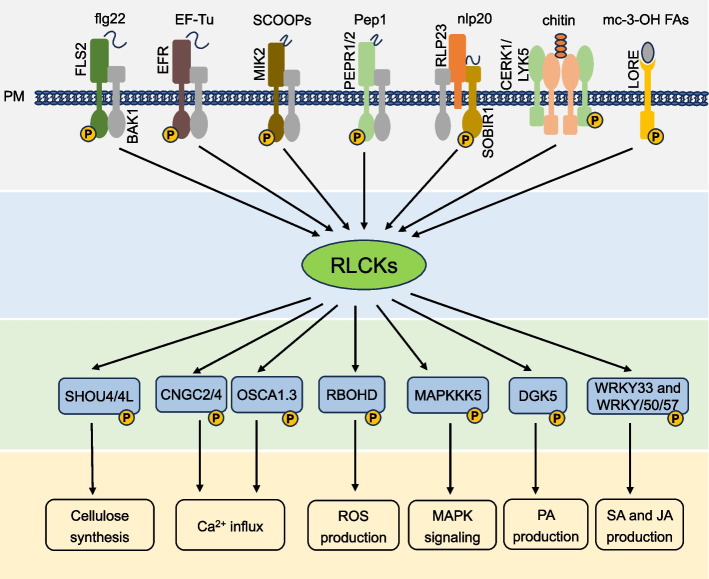
Roles of RLCKs on signal convergence and divergence during PTI. Signals from PRRs are converged on RLCKs and then diverged to the downstream signals, such as SHOU4/4L, CNGC2/4, OSCA1.3, RBOHD, DGK5, MAPKKK5 phosphorylations. Solid arrows indicate positive regulations. BAK1: brassinosteroid insensitive1-associated kinase1; BIK1, Botrytis-induced kinase1; BRs: brassinosteroids; BRI1: brassinosteroid insensitive 1; CERK1: chitin elicitor receptor kinase 1; CNGC: cyclic nucleotide-gated channel; EF-Tu: elongation factor-thermal unstable; EFR: EF-Tu receptor; flg22: flagelin22; FLS2: flagellin-sensing2; LORE: lipooligosaccharide-specific reduced elicitation; LYK5: lysin motif receptor kinase5; MAPK: mitogen-activated protein kinase; mc-3-OH FAs: medium-chain 3-hydroxy fatty acids; MIK2: MDIS1-interacting receptor-like kinase2; OSCA: hyperosmolality-gated calcium-permeable channel; PA: phosphatidic acid; PM: plasma membrane; PTI: pattern-triggered immunity; RBOHD, respiratory burst oxidase homolog D; RLCK: receptor-like cytoplasmic kinase; RLP23: receptor-like kinase23; SCOOP: serine rich endogenous peptide; SOBIR1: suppressor of brassinosteroid insensitive1. WRKY33/50/57: WRKY transcription factors

Plant RLCKs regulate various biological processes, including response to biotic and abiotic stresses, hormone signaling, sexual production, and additional vital functions (Jurca et al. 2008; Lin et al. 2013a; Tanaka et al. 2012; Hirano et al. 2015; Hailemariam et al. 2024). RLCKs transduce receptor-mediated signaling by coupling with RLKs/RLPs to relay cellular signaling via phosphorylation which forms a ligand-RLK-RLCK intertwined network to regulate various cellular events (Lin et al. 2013a; Liang and Zhang 2022; Liang and Zhou 2018; Gao et al. 2023). For example, the RLK flagellin-sensing 2 (FLS2) recognizes the bacterial flagellin or the pattern peptide flg22 to induce PTI. Upon perception, flg22 induces the interaction of FLS2 with its coreceptor, BRI1-associated kinase 1 (BAK1) to form FLS2-BAK1 receptor complex (Chinchilla et al. 2007). As soon as flg22 binds to the complex, trans-phosphorylation occurs between FLS2 and BAK1 which leads to activation of the PRR complex and recruits RLCKs, including RLCK-VII member Botrytis-induced kinase 1 (BIK1). Activated PRR complex phosphorylates BIK1 which transduces signal to downstream components to activate PTI responses (Gómez-Gómez and Boller 2000; Jelenska et al. 2017). BIK1 directly interacts with and phosphorylates RBOHD, Ca^2+^ channels, diacylglycerol kinase 5 (DGK5), and SHOU4/4L to activate ROS production, Ca^2+^ influx, PA production, and cell wall synthesis respectively (Li et al. 2014; Kadota et al. 2014; Kong et al 2024; Thor et al. 2020; Wang et al. 2019, 2023). Thus, BIK1 connects PAMP perception to multiple immune responses (Fig. 1). AvrPphB susceptible1 (PBS1)-Like 1(PBL1), the closest homologue of BIK1, functions redundantly with BIK1. Downstream FLS2-BAK1 complex, two RLCK members BSK1 and PBL19 interact with and phosphorylate MAPKKK5 to activate MAPK cascades (Yan et al. 2018; Dong et al. 2023).

The signaling of flg22 perception by FLS2 demonstrates a prevailing paradigm in PTI immune responses. The perception of bacterial elongation factor thermo unstable (EF-Tu) by EF-Tu receptor (EFR) is largely overlapped with that of flg22 by FLS2. Besides the responses mentioned in FLS2 signaling, EFR induces BIK1 relocalization to nucleus where it interacts with and phosphorylates WRKY transcription factors WRKY33 and WRKY50/57 to regulate salicylic acid (SA) and jasmonic acid (JA) synthesis (Lal et al. 2018). Similarly, Arabidopsis responds to DAMP peptide Pep1 and endogenous phytocytokine Serine-rich endogenous peptides (SCOOPs) by Pep1 receptor (PEPR1/2) and Male discoverer 1-interacting receptor-like kinase 2 (MIK2) in the same way. BIK1 and PBL1 transduce PRR signal to downstream thus to activate PTI. The flg22 triggered PTI signaling is conserved in rice, OsFLS2 associates with coreceptor OsSERK2 to activate downstream OsRLCK176/185, resulting in PTI responses (Zhao et al. 2024a, b).

In fact, more and more findings show that variable sets of RLCKs mediate different PRR signaling. BIK1, PBL1, BSK1, RIPK, PBL19, PBL20, PBL13, CDG1, and PTI compromised receptor-like cytoplasmic kinase (PCRK)1/2 are involved in FLS2/EFR mediated immune signaling. BIK1, PBL1, BSK1, RIPK, PBL19, PBL20, PCRK1/2, and PBL37-PBL40 are involved in chitin-triggered immunity in Arabidopsis. Rice RLCK members OsRLCK57, OsRLCK107, OsRLCK118, OsRLCK176, OsRLCK185, and OsRLCK278 function downstream of rice chitin elicitor receptor kinase 1 (OsCERK1) in peptidoglycan (PGN) and chitin triggered immunity (Ao et al. 2014; Li et al. 2017; Kanda et al. 2017). The oomycete PAMP nlp20 forms a complex with the leucine-rich repeat receptor protein (LRR-RP) RLP23, LRR-RKs SOBIR1 and BAK1 to mediate NLP-triggered immunity which depends on RLCK PBL30 and PBL31 (Albert et al. 2015; Pruitt et al. 2021). Arabidopsis RLP42 recognizes the fungal protein SCFE1 from *Sclerotinia sclerotiorum*, by associating with two co-receptors SOBIR1 and BAK1 and recruiting BIK1 to the RLP42-SOBIR1-BAK1 complex to transmit immune signals initiated by SCFE1 recognition (Zhang et al. 2020). Lipooligosaccharide-specific reduced elicitation (LORE), a G-type lectin receptor kinase, senses bacterial lipooligosaccharide (LPS) or medium-chain 3-hydroxy fatty acid (mc-3-OH-FA), to trigger immune responses depends on RLCK PBL34, and its paralogs PBL35 and PBL36 (Luo et al. 2020).

RLCKs relay signals from RLK/RLP perception to downstream responsive factors, not only in plant immune responses but also in other processes. Thus, RLCKs are believed to be the central players indispensable for receptor mediated signaling (Liang and Zhou 2018). Constitutive activation of plant immunity is harmful to plants, PRRs mediated immune signaling must be under tight control. RLCK is one of the hotspots for regulation. The regulation of RLCK signaling has been reviewed elegantly elsewhere (Liang and Zhang 2022). Since massive RLCKs are encoded in plant genomes, RLCKs orchestrate plant cellular communications in various processes which has also been summarized (Hailemariam et al 2024). This review examines the roles of RLCKs in various plant immune responses and crosstalk in between, highlighting their pivotal function in signal convergence and divergence, which are critical for the regulation of plant immunity.

### RLCK mediated signal convergence and divergence in PTI

RLCKs act as hubs to transduce a common set of downstream signaling activated by plant PRRs. For instance, BIK1 works as one important node for signal convergence, receiving signals from various upstream receptors perceiving different PAMPs during plant immune responses. BIK1 mediated signal convergence interprets overlapping immune responses triggered by different PRRs, as revealed by transcriptome analysis (Bjornson et al. 2021). On the other hand, differential phosphorylation events of RLCKs by the upstream RLKs/RLPs may result in differentiated RLCK modifications for regulating different downstream signaling events (Tang et al. 2017). BIK1 also mediates divergent downstream signaling to activate a repertoire of immune responses, including ROS production, Ca^2+^ influx, MAPK signaling, cellulose synthesis, PA production, plant hormone signaling, and others (Fig. 1). In addition to BIK1, other RLCK members also transduce convergent signals and mediate divergent responses, such as PBL1, BSK1, and RIPK. As mentioned above, the combinations of different RLCK sets are involved in different PRR signaling which help explain the variations between PAMPs. The overlapping and differential regulation of cellular signaling suggests the complexity of RLCKs for plant immunity.

### Controlling of ROS production

RBOHs, also known as NADPH oxidases, are major ROS-generation enzymes during pathogen infections. RBOHs transfer electrons from cytosolic NADPH to FAD, then to apoplastic oxygen to form superoxide anion radical which convert to hydrogen peroxide (Kaur et al. 2014). AtRBOHD has been identified as the first substrate to be phosphorylated by RLCKs (Kadota et al. 2015). RLCKs play important roles in activating ROS production during plant immunity (Lee et al. 2020).

RLCKs phosphorylate to activate RBOHD and regulate ROS production in PTI (Kadota et al. 2014; Li et al. 2014). Arabidopsis RBOHD was shown to be the master NADPH oxidase that controls ROS production in PTI response. *rbohD* demonstrates abolished ROS production. RBOHD was identified in the PRR complexes of EFR and FLS2, suggesting direct regulation by the immune receptor complex. BIK1, and its closest homologue PBL1, interacts with and phosphorylates RBOHD at multiple residues on N-terminal domain, including Ser39, Thr123, Ser339, Ser343, and Ser347 (Kadota et al. 2014). Abolishment of phosphorylation residues leads to impaired ROS production, suggesting a crucial role of phosphorylation on RBOHD activity control (Kadota et al. 2014). RBOHD activation also requires Ca^2+^ binding. Calcium channel blocker or Ca^2+^ chelator treatment suppresses RBOHD activity (Kadota et al. 2004). Phosphorylation of RBOHD is a prerequisite of Ca^2+^-dependent activation which is consistent with the fact that Ca^2+^ binding motifs (EF-hand) are present on N-terminus of RBOHD (Kimura et al. 2012). The RLCK-VII subfamily member RPM1-induced protein kinase (RIPK) is also involved in RBOHD-mediated ROS production. Lipopolysaccharides (LPS) or its core structure Lipid A induced a biphasic ROS production in Arabidopsis, which is dependent on RIPK. Similar to BIK1, RIPK directly interacts with and phosphorylates RBOHD at several residues on N-terminus, including the crucial residues Ser343 and Ser347 (Li et al. 2021; Liu et al. 2021). The conserved MAP4 kinase SIK1 regulates RBOHD activity in BIK1 dependent and independent ways. Firstly, SIK1 associates with, phosphorylates, and stabilizes BIK1 thus activating RBOHD. Second, SIK1 directly binds to and phosphorylates RBOHD to enhance ROS production. Upon PAMP treatment, SIK1 phosphorylates RBOHD at four Serine residues on N-terminus, Ser8, Ser9, Ser339, and Ser347 (Zhang et al. 2018). In contrast, Arabidopsis RLCK-VII member PBL13 negatively regulates flg22-triggered ROS burst by directly phosphorylating RBOHD at Ser862 and Thr912 on the C-terminus, affecting RBOHD activity and stability respectively (Lee et al. 2020; Lin et al. 2015).

The regulation of ROS production seems conserved. In rice, two RLCK-VII subfamily members OsRLCK176 and OsRLCK118 phosphorylate OsRbohB on the N-terminus to regulate ROS production, suggesting similar regulation of RBOH proteins by RLCKs across species (Fan et al. 2018). OsRBOHI is recently reported to be the key enzyme for ROS production in rice. Upon PAMP treatment, OsRBOHI was phosphorylated at 14 residues on N-terminus, including 6 conserved residues, Ser38, Ser144, Ser170, Ser346, Ser350, and Ser354 (Zhao et al. 2024a, b). The last three correspond to the crucial residues Ser339, Ser343, and Ser347 on Arabidopsis RBOHD. Whether the phosphorylation of OsRBOHI is mediated by RLCKs needs further investigation. The four closest homologues of Arabidopsis BIK1, OsRLCK57, OsRLCK107, OsRLCK118, and OsRLCK176 have been reported to regulate chitin- and PGN-induced ROS generation (Li et al. 2017). In the liverwort *Marchantia polymorpha*, RLCK MpPBLa directly phosphorylates MpRBOH1 at specific and conserved residues to regulate ROS production, suggesting the conservation of the RLCK-RBOH module during PTI in land plants (Chu et al. 2023).

Besides BIK1, RIPK, and PBL13 which directly interact and phosphorylate RBOHD to regulate activity, additional RLCK members also participate in ROS production control in unclear ways. PCRK1 is also required for the flg22-induced ROS burst (Kong et al. 2016; Sreekanta et al. 2015). RLCK-XII subfamily members BR signaling kinase1 (BSK1) is required for ROS production (Shi et al. 2013; Yan et al. 2018). Arabidopsis RLCK-VII member PBL30 and PBL31 are responsible for ROS generation downstream of RLP23 (Pruitt et al. 2021). Actually, RLCKs from multiple subfamilies are generally involved in ROS production. RLCK members from VII-5, -7, and -8 subgroups are shown to regulate ROS production induced by flg22, elf18, and chitin. While those from VII-4 are specifically involved in chitin-induced ROS production (Rao et al. 2018). In contrast, Constitutive differential growth1 (CDG1) negatively regulates flg22 and chitin-induced ROS by promoting the degradation of FLS2 and CERK1 (Yang et al. 2021). Like in Arabidopsis, members of the rice RLCK VII-4 subgroup are required for flg22 and chitin-induced ROS production and other PTI responses (Jalilian et al. 2023). Rice *broad-spectrum resistance 1* (*OsBSR1*) encodes the receptor-like cytoplasmic kinase OsRLCK278 which is responsible for chitin-induced ROS production (Kanda et al. 2017). In *N. benthamiana*, members of RLCK-VII-6, -7, and -8 subgroups play an important role in regulating flg22- and chitin-induced ROS production, similar to those in Arabidopsis and in rice (Huang et al. 2024) differentially regulate the Avr4/Cf-4-triggered biphasic burst of reactive oxygen species The tomato RLCK-VIII subfamily Pto-interacting 1 (PTI1) is responsible for flg22-induced ROS generation and resistance against pathogen infection. It is hypothesized that PTI1 might phosphorylate RBOH and thereby contribute to its activation for ROS production (Schwizer et al. 2017). The findings suggest a complicated regulation of RBOH by RLCKs in ROS production.

### Regulation of Ca^2+^ influx

Calcium participates in diverse signaling pathways to regulate plant growth, development, and immunity (Köster et al. 2022). Rapid calcium influx can be triggered by PAMPs after being perceived by PRRs. The elevation of cytoplasmic calcium concentration could activate calcium-dependent protein kinases (CDPKs or CPKs), which depend on calcium ions to activate kinase activity to trigger the downstream immune responses (Boudsocq et al. 2010). RLCKs are essential for converging signals on calcium channels for modulating Ca^2+^ fluxes.

Cyclic nucleotide-gated channels (CNGCs) are nonselective cation channels that are opened by the direct binding of cyclic nucleotide. The regulation of calcium channels by RLCKs has emerged as a significant area of research to elucidate plant innate immunity (Köster et al. 2022; Talke 2003; DeFalco and Zipfel 2021). OsCNGC9 has been identified as a calcium-permeable cation inward channel to mediate PAMP-induced signaling in rice. Upon infection, OsRLCK185 phosphorylates the C-terminus of OsCNGC9 to activate the channel activity, facilitating Ca^2+^ influx and downstream immune responses (Wang et al. 2019). Rice OsCPK17 is rapidly induced upon PAMP stimulation to phosphorylate OsRLCK176 at Ser32, Ser83, and Ser209, suggesting the relation between Ca^2+^, CPK, RLCK, and plant immunity (Mou et al. 2024).

The *Arabidopsis* RLCK-VII BIK1 and PBL1 are required for Ca^2+^ influx upon flg22 treatment (Ranf et al. 2014). The *bik1* and *pbl1* mutants showed an impaired Ca^2+^ influx upon flg22 treatment, suggesting the importance of RLCK-VII BIK1 and PBL1 in the regulation of Ca^2+^ influx (Li et al. 2014; Ranf et al. 2014). Besides, AtCNGC2 and AtCNGC4 together generate a calcium channel, which is blocked by calmodulin at the resting state but activated by PAMP induction (Tian et al. 2019; Wang et al. 2017a, b). BIK1 phosphorylates and activates the CNGC2/4 channel to increase the cytoplasmic Ca^2+^ concentration upon pathogen infections (Tian et al. 2019). Ca^2+^ influx is both positively and negatively regulated to maintain a proper concentration of cytoplasmic Ca^2+^ concentration. *Arabidopsis* calmodulin 7 (CAM7) interacts with the CAM-binding domain and the isoleucine-glutamine (IQ) domain of CNGC2 and CNGC4 which negatively regulates CNGC2/4 channel activity and flg22-induced PTI (Tian et al. 2019). Besides, another pair of CNGC channels CNGC20/CNGC19 is involved in Ca^2+^ transport in plant immunity. CNGC20 is also under the control of BIK1 which interacts with and stabilizes CNGC20. Mutation of CNGC20 (*cngc20-4*) leads to enhanced PTI responses and ETI hypersensitive cell death, suggesting a negative role in regulation of PTI and ETI. In a genetic screening, *cngc20* was identified to suppress *bak1serk4* cell death. BAK1 directly interacts with and phosphorylates CNGC20 at specific sites in the C-terminal domain, which enhances CNGC20 stability. CNGC20/CNGC19 functions as a hyperpolarization-activated Ca^2+^-permeable channel whose homeostasis is critical in cell death control (Yu et al. 2019; Zhao et al. 2021).

Reduced hyperosmolality-induced Ca^2+^ increase channel (OSCA) is another family of Ca^2+^-permeable channels that are involved in plant immunity. In *Arabidopsis*, OSCAs contain nine transmembrane helices, where the N terminus is short and the C terminus is large (Yuan et al. 2014). OSCA1.3 and OSCA1.7 were found to function in stomatal immunity during elicitor treatment (Thor et al. 2020). Upon flg22 treatment, BIK1 rapidly phosphorylates the N-terminal loop of OSCA1.3 at Ser49, Ser50, and Ser54 (predominantly at Ser54) (Thor et al. 2020). Besides, PBL1 could also phosphorylate OSCA1.3 at Ser54 during immune signaling (Thor et al. 2020). BIK1, together with PBL1, is genetically involved in Ca^2+^ influx upon MAPM perception (Li et al. 2014; Ranf et al. 2014). The phosphorylated serine with the motif (Ser-X-X-Leu) is present in both RBOHD and OSCA1.3, implying a synergistic regulation of RBOHD and OSCA1.3 by BIK1 (Thor et al. 2020).

### Modulation of MAPK signaling pathway

RLCKs can directly regulate the MAPK signaling pathway (Zhou et al. 2019; Cui et al. 2018). Upon infection, plants physiologically change to adapt and defend pathogen attacks which rely on transcriptional remodeling initiated by PRRs. Transcriptional remodeling works downstream of MAPK cascades. The MAPK cascades include MAPKKK, MAPKK, and MAPK, which are conserved in eukaryotes (Zhang and Klessig 2001). The MAPK signaling pathways are typically initiated by the activation of MAPKKK through the interaction with a small GTPase or the phosphorylation by protein kinase downstream from cell surface receptors (Zhang and Liu 2002). MAPKKK then phosphorylates MAPKK at the threonine and/or tyrosine residue sites, activating MAPKK, which subsequently activates the MAPK (Haddad et al. 2003). MPK3, MPK4, and MPK6 are rapidly phosphorylated upon PAMP induction which are differentially activated by upstream MAPK kinases. MPK4 is activated by MEKK1 and MKK1/2 while MPK3 and MPK6 are activated by MAPKKK3/5 and MKK4/5 (Asai et al. 2002; Bi et al. 2018; Gao et al. 2008; Qiu et al. 2008; Suarez-Rodriguez et al. 2007; Yan et al. 2018). How PRRs transduce signals to MAPKKKs during plant immune responses has been extensively investigated.

Arabidopsis RLCK VII-4 members are required for chitin-triggered activation of the MAPK signaling pathway, mediating the CERK1 signaling (Rao et al. 2018). Specifically, chitin-triggered MAPK activation was impaired in the *rlck vii-4* mutant plants (Rao et al. 2018). As the primer receptor for chitin, AtLYK5 forms a chitin-induced complex with AtCERK1 (Cao et al. 2014). The Arabidopsis RLCK PBL27 and BSK1 directly interact with and phosphorylate MAPKKK5 to transduce immune signals from the chitin receptor complex (CERK1-LYK5) and flagellin receptor complex (FLS2-BAK1), respectively, to MAPK cascades (Yan et al. 2018; Yamada et al. 2016). The RLCK VII-4 members, including PBL19, strongly interact with MAPKKK5 and phosphorylate the C-terminal region of MAPKKK5 to enhance plant immunity (Dong et al. 2023). The interactions between RLCKs and MAPKKKs and the subsequent activation of MAP kinase cascades require the λ and κ isoforms of 14–3-3 proteins, which working as a scaffold directly interact with multiple RLCKs and MAPKKKs. RLCKs access to the C-terminus of MAPKKK5 when 14–3-3 protein release the auto-inhibition between N- and C-termini of MAPKKK5 and exert the kinase activity to phosphorylate the latter (Dong et al. 2023). In brief, RLCK VII-4 subfamily members transduce activation signal from PRR to MAPKKK3/5 to activate MPK3 and MPK6. In addition, MPK4 is activated by MEKK1 when its Ser603 is phosphorylated upon PAMP induction (Bi et al. 2018). In tomato, 14–3-3 proteins TFT1 and TFT3 associate with both the Pto/Pto resistance and fenthion sensitivity (Prf) NLR resistance complex and MAPKKKα, mediating the activation of MAPK cascade by Pto/Prf complex (Sheikh et al. 2023). As will be discussed later, Pto is a RLCK which involved in ETI. RLCK-mediated MAPK activation demonstrates a PRR- or PAMP-dependent manner. RLCK-VII-4 subgroup (PBL19, PBL20, PBL37, PBL38, PBL39 and PBL40) is specifically required for chitin-triggered MAPK activation while the RLCK-VII-8 subgroup (BIK1, PBL1, PBL11, PBL9, and PBL10) is specifically for Pep2-induced MAPK activation, suggesting differential responses between treatments (Rao et al. 2018). Additional two RLCK VII-4 subfamily members PCRK1/PCRK2 also mediate MAPK activation during PTI (Kong et al. 2016; Yamada et al. 2016).

In rice, downstream of chitin receptor OsCERK1, OsRLCK185, the closest rice homologue of Arabidopsis PBL27, interacts with and phosphorylates the C-terminal regulatory domain of OsMAPKKKε to activate the MAPK signaling cascade and positively regulate rice immunity (Wang et al. 2017a, b). Like that in Arabidopsis, members from RLCK-VII-4 subgroup are required for MAPK activation in rice (Jalilian et al. 2023). These findings demonstrate an important and conserved role of RLCKs in linking PRRs to MAPK cascades during plant immunity.

### Regulation of PA production

Recently, BIK1 was shown to regulate PA production, together with MPK4. Not only as structural components of cell membranes, PA also functions as a universal second messenger mediating multiple cellular signaling events. For example, PA modulates ROS production by binding to RBOHD N-terminus. PA probably plays important roles in plant immune responses since rapid PA production, or PA burst, were observed in tomato and rice suspension cells treated with PAMPs. Arabidopsis BIK1 interacts with and phosphorylate DGK5 at Ser506 to activate its enzyme activity, leading to a rapid PA burst. Since PA binds and stabilizes RBOHD, BIK1 could indirectly regulate ROS production and plant immunity by DGK5 phosphorylation-induced PA production (Kong et al. 2024). In contrast, PRR-activated MPK4 interacts and phosphorylates DGK5 at Thr446, which suppresses DGK5 activity and PA production, resulting in attenuated plant immunity. DGK5 is involved both in plant PTI and in ETI, suggesting the importance of PA in plant defense. BIK1 and MPK4 regulate DGK5 activity inversely, suggesting balanced PA production is critical for plant immunity (Kong et al. 2024).

### Involvement in cellulose synthesis

RLCKs are involved in cellulose synthesis. The cell wall is a complex polysaccharide network that provides a physical barrier against pathogen attack. To overcome this barrier, pathogens secrete cell wall-degrading enzymes to break down the cell wall components, including cellulose. As an immune response to microbial attacks, plants have adapted to recognize cellulose degradation products (DAMP) and to reinforce or remodel the cell wall (Molina et al. 2024). On flg22 perception, the FLS2-BAK1 complex phosphorylates BIK1 which could interact with and phosphorylate SHOU4/4L to regulate cellulose synthesis complexes (CSCs) exocytosis (Polko et al. 2018; Wang et al. 2023). BIK1 phosphorylates SHOU4L at multiple residues, including Ser7, Ser11, Ser45, Ser64, Ser95, Ser111, Ser156, and Ser169. Being phosphorylated, the activity of SHOU4/4L is inhibited and the CSCs move to the plasma membrane for cell wall synthesis (Wang et al. 2023). The involvement of BIK1 in SHOU4/4L-mediated cellulose synthesis implies the role of plant RLCK in cell wall remodeling and cell wall-mediated immunity.

### Modulation of plant hormone signaling

Multiple plant hormones are closely related to plant immunity, especially SA, JA, ABA, and ethylene. RLCKs play important roles in plant hormones signaling and hormone-mediated immunity.

Salicylic acid (SA) mediates plant defense by inducing local defense and systemic acquired resistance (SAR) (Li et al. 2022). PCRK1 and PCRK2 function as key regulators of SA biosynthesis. *pcrk1 pcrk2* double mutant demonstrates reduced SA level which is attributed to reduced induction SA biosynthesis genes, *SARD1*, *CBP60g*, and *ICS1* (Kong et al. 2016). Since PCRK1 and PCRK2 work downstream of PRR, they respond to PAMP perception to induce SA biosynthesis. In addition, the BIK1 is required to defend *Botrytis cinerea* infection depends mostly on SA levels, suggesting a role of the RLCK in regulating SA biosynthesis and signaling during plant immune responses (Veronese et al. 2006). Responding to EF-Tu, BIK1 is uniquely phosphorylated by EFR and localizes to the nucleus where it interacts with WRKY transcription factors WRKY33 and WRKY50/57 to regulate SA and JA accumulation during plant immunity (Lal et al. 2018).

Jasmonic acid (JA) affects plant growth and development by mediating plant responses to biotic and abiotic stresses (Ruan et al. 2019). The tomato receptor-like cytoplasmic kinase, SlZARK1, regulates wound-induced JA accumulation, suggesting RLCKs function as a new component during wound-induced response (Sun et al. 2021).

Ethylene (ET) is a gaseous phytohormone that responses to stresses (Binder 2020). BIK1 regulates ET signaling by interacting with PEPR1, the receptor of *Arabidopsis* Pep1, of which the family members are DAMPs (Liu et al. 2013). The *bik1* and *pepr1*/*pepr2* mutant seedlings were partially insensitive to ET, suggesting that BIK1 and PEPRs mediate ET-mediated plant immunity (Liu et al. 2013).

ABA regulates various cellular events, such as seed maturation and dormancy, plant growth and flowering, and resistance to abiotic stresses (Finkelstein et al. 2002). *ABA- and osmotic-stress-inducible receptor-like cytosolic kinase 1* (*ARCK1*) encodes an RLCK which interacts with and undergoes phosphorylation by CRK36 transducing signals in response to ABA during post-germinative growth (Tanaka et al. 2012).

Brassinosteroids (BRs) are polyhydroxylated steroidal hormones which regulates plant growth and development, as well as plant defense (Yang et al. 2011). BRs are perceived by the extracellular domain of RLK brassinosteroid insensitive 1 (BRI1) to induce BRI1-BAK1 interaction and activation of BRI1 (Liu et al. 2011). On perception, BRI1-BAK1 complex phosphorylates the RLCK-XII BSKs to activate BR signaling and responses, including root development, pollen development, cellulose biosynthesis, and others (Tang et al. 2008). Besides, BRI1 directly phosphorylates BIK1 to transduce BR signaling (Lin et al. 2013b).

## RLCKs function in NLR-mediated signaling

RLCKs also participate in NLR-mediated signaling. NLRs, which contain either a coiled-coil domain (CC-NLR) or a Toll-interleukin-1 receptor domain (TIR-NLR), sense pathogen effectors to initiate ETI (Bentham et al. 2018; Huang et al. 2023).

The ZAR1 resistosome is a newly discovered pentameric calcium-permeable channel that induces programmed cell death upon infection of avirulent pathogens. ZAR1 is an ancient CC-type NLR that originated before the split of eudicots and monocots and is conserved across plant classes. Arabidopsis PBL2 is a member of Group-VII RLCK which is uridylylated by *Xanthomonas campestris* type III secreted effector (T3SE) AvrAC. Uridylylated PBL2 is specifically recruited to the preformed ZAR1-RKS1 complex to form functional ZAR1 resistosome. Thus, PBL2 functions as a decoy substrate of T3SE, which is monitored by ZAR1. Noteworthy, though lacking of kinase activity, pseudokinase RKS1 belongs to the RLCK-XII subfamily (Gong et al 2022; Wang et al. 2015; Bi et al. 2021; Adachi et al. 2023). Arabidopsis pseudokinase HopZ-ETI-deficient1 (ZED1) is a homologue of RKS1 and belong to RLCK XII subfamily. ZED1 is another decoy monitored by ZAR1. ZED1 interacts with effector HopZ1a and is acetylated on Thr125 and Thr177 which activates ZAR1 resistance complex, resulting in ETI activation (Lewis et al. 2013). Two RLCK-VII members Suppressor of ZED1-D1 (SZE1) and SZE2 interact with ZED1 and ZAR1 and are required for the ZED1-activated autoimmune response and HopZ1a-triggered immunity (Liu et al. 2019). Similarly, the *N. benthamiana* RLCK subfamily -VII member XopJ4 Immunity 2 (JIM2) functions together with NbZAR1 to recognize the T3SE XopJ4 from *Xanthomonas perforans.* Mutation of JIM2 leads to impairment of XopJ4 recognition by NbZAR1 (Schultink et al. 2019). The examples suggest that ZAR1 functions together with RLCK to detect T3SE and activate ETI (Martel et al. 2020).

The Arabidopsis RLCK subfamily -VII member PBS1 functions as a decoy to lure *P. syringae* effector AvrPphB which is a cysteine protease. The integrity of PBS1 is monitored by the CC-NLR resistance protein Resistance to *Pseudomonas syringae* 5 (RPS5). PBS1 interacts with RPS5 on its CC domain in the rest state. Upon infection, PBS1 is cleaved by AvrPphB which results in the release of auto-inhibition of RPS5 thus triggering ETI (Ade et al. 2007). Another RLCK subfamily - VII member RIPK interacts with and phosphorylates RPM1-interacting 4 (RIN4), contributing to the NLR-mediated defense response against *P. syringae* (Liu et al. 2011). RIN4 phosphorylation by RIPK depends on *P. syringae* effector AvrB and AvrPRM1 which is monitored by the Arabidopsis NLR protein RPM1. Though RIPK is not a decoy in this case, it acts as a trigger to modify decoy RIN4 (Chung et al. 2011; Liu et al. 2011). RLCK CDG1 negatively regulates Arabidopsis PTI and is involved in AvrRpm1-induced RIN4 phosphorylation (Yang et al. 2021).

In tomato, a couple of RLCK members are also involved in NLR-mediated signaling and resistance. *Pto,* which encodes an RLCK, is the first disease-resistance gene cloned from a plant that confers race-specific resistance. Pto directly interacts with *P. syringae* type III effectors AvrPto and AvrPtoB to initiate ETI through the NLR protein Prf (Kim et al. 2002; Scofield et al. 1996; Tang et al. 1996). Tomato RLCK Avr9/Cf-9 induced kinase 1 (ACIK1) is required for resistance protein Cf9 and Cf4-mediated resistance (Rowland et al. 2005). In addition, members of RLCK-VII-7 subgroup also play an important role in the Avr4/Cf-4-induced ETI and in the resistance of *N. benthamiana* to *Phytophthora palmivora* (Huang et al. 2024). Tomato Mai1 (SlMai1) is a homologue of Arabidopsis BSK1 with 80% amino acid identity and belongs to the RLCK subfamily -XII. SlMai1 interacts with tomato MAPKKKα, positively regulating tomato PCD induced by several CC-NLR proteins in a manner of kinase activity-dependent. SlMai1 acts between CC-NLR proteins and the MAPK signaling cascade. Virus-induced gene silencing of Mai1 homologs in *N. benthamiana* leads to compromised PCD induced by four CC-NLR proteins including Prf, indicating the important roles of RLCK in NLR-mediated resistance (Roberts et al. 2019).

**Fig. 2:**
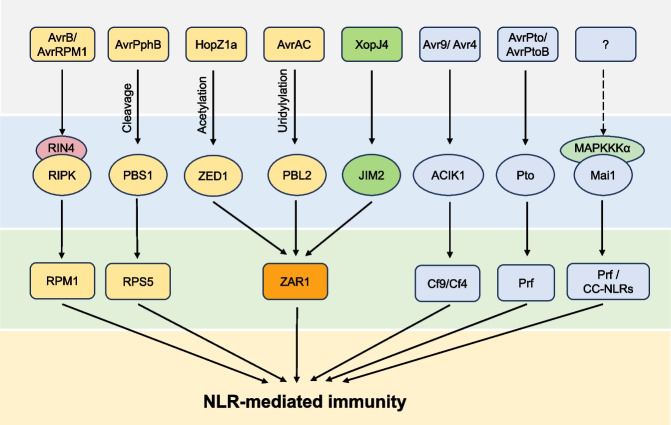
Roles of RLCKs in relation with NLR-mediated ETI. RLCKs are targeted by pathogen effectors and monitored by NLRs to mediate ETI. Solid arrows indicate positive regulations; dashed lines indicate unelucidated regulations. Effectors include AvrB/AvrRPM1, AvrPphB, HopZ1a, AvrAC, XopJ4, Avr9/Avr4, and AvrPto/AvrPtoB. RLCKs include ACIK1, JIM2, Mai1, PBL2, PBS1, Pto, RIPK, and ZED1. NLRs include CC-NLR, Cf9/Cf4, Prf, RPM1, RPS5, and ZAR1. ACIK1: AVR9/CF-9 induced kinase1; JIM2: XopJ4 immunity 2; PBL: AvrPphB susceptible 1 (PBS1)-like; Prf: PTO resistance and fenthion sensitivity; RIPK, RPM1-induced protein kinase; MAPKKK: mitogen-activated protein kinase kinase kinase; NLR: nucleotide-binding domain and leucine-rich repeat containing; ZAR1: HopZ-activated resistance1; ZED1: HopZ-ETI-deficient1

Altogether, RLCKs play versatile roles in NLR-mediated effector recognition and ETI activation which suggests a convergent signaling mediated by RLCK (Fig. 2).

### RLCKs are hot spots of effector target

RLCKs are frequently targeted by pathogen effectors for pathogen virulence and host immunity suppression, suggesting the pivotal roles that RLCKs exert in plant immunity. For example, RLCK- VII subfamily member BIK1 is targeted by the fungal effector NIS1 (necrosis-inducing secreted protein1), which is broadly conserved in filamentous fungi, to inhibit the kinase activity and subsequent immune signaling (Irieda et al. 2019). RLCKs BIK1, PBL8, and PBL17 are targeted by the oomycete effector RXLR25 from *Phytophthora capsici* to inhibit their phosphorylation and the downstream immune responses (Liang et al. 2021). OsRLCK185 is targeted by the *Xanthomonas oryzae* effector Xoo1488, resulting in the suppression of chitin-triggered immunity (Yamaguchi et al. 2013). The wheat RLCK-VII subfamily member TaPsIPK1 is targeted by the rust effector PsSpg1, enhancing the kinase activity of TaPsIPK1 for TaCBF1d phosphorylation which (Wang et al. 2022).

Since playing important roles in plant immunity and are hotspots of effector targeting, RLCKs are also deployed for monitoring pathogen infection. As mentioned above, PBS1 and PBL2 function as decoys in the presence of corresponding resistance proteins and are targets of *P. syringae* effector AvrPphB and *X. campestris* effector AvrAC. Both play critical roles in activating plant immunity. PBS1 and its homologues, BIK1, PBL1, and PBL2, are targeted by AvrPphB to result in cleavage and suppression of PTI (Zhang et al. 2010). PBL2, together with BIK1, PBL1, and RIPK, are targeted by AvrAC for uridylylation on the kinase activation loop which impairs kinase activity and suppresses PTI (Feng et al. 2012; Wang et al. 2015).

### RLCKs mediated crosstalk between PTI and ETI

Recent discoveries demonstrate a firm connection between PTI and ETI. PTI is required for ETI, while ETI enhances PTI. Mutation of either PRRs or RBOHD leads to impaired ETI responses upon incompatible infection. Noteworthy, *bik1* mutants were also compromised in ETI-mediated bacterial resistance. Likewise, ETI enhance PTI by inducing gene expression of PTI components, including PRRs, RBOHD, and BIK1. Besides, ETI components are also required for PTI responses, such as PAD4, EDS1, and helper NLR ADR1. During plant immunity, PTI and ETI collaborate to achieve robust resistance by triggering shared downstream defense responses, though different strength, suggesting common nodes are involved in activating defense signaling (Yu et al. 2024). For example, BIK1 has many targets in PTI while many ETI-triggered responses are *BIK1*-dependent, indicating the dual roles of BIK1 during both PTI and ETI (Yuan et al. 2021). On the other side, the decoy RLCKs PBS1 and PBL2 are involved in PTI activation. Mutation of them suppress PTI responses (Zhang et al 2010; Wang et al 2015). Both RIPK and CDG1 are involved in RIN4-mediated ETI and in PTI regulation (Li et al. 2021; Yang et al. 2021). Thus, RLCKs also play a role during PTI and ETI crosstalk.

## Conclusions and perspectives

Cell surface receptors, mostly RLK, are central to plant adaptation to the environment, sexual reproduction, growth, and development. RLCKs play pivotal roles to transduce perception signals from receptors, including those from PRRs. An array of PRRs have been identified from various plants. Despite differences in structure and ligand, the immune responses they initiated are largely overlapping, suggesting similar signaling pathways shared (Bjornson et al. 2021). The PRR-mediated signaling pathways form an intertwined network, with crucial convergent and divergent RLCK hubs, including BIK1. The convergent signaling mediated by BIK1, and other RLCK members, could interpret the overlapping responses. Noteworthy, the immune responses also show differences between PAMPs, flg22 and nlp20, for example (Wan et al. 2019). The differential responses suggest variable sets of RLCKs, or divergent roles of RLCKs, are involved in specific PRR signaling. BIK1 mediates divergent signaling which is consistent with its versatile roles in plant immunity, demonstrated by modulating multiple immune components, including RBOHD, CNGC2/4, OSCA1.3, DGK5, and SHOU4/4L.

The identified PRRs and RLCKs are the tip of an iceberg, and a summary of the current findings about RLCKs is underrepresented. RLCKs comprise one of the largest gene families in plant genomes, with 149 RLCKs in Arabidopsis and 379 RLCKs in rice (Shiu et al. 2004; Vij et al. 2008). Up to date, only limited RLCK members are well-characterized (Supplementary Table 1). PRRs are also underestimated. LRR-RLK/RLP and NLR gene families show concerted expansion and contraction of the size of their repertoires across plant species, suggesting more PRRs are encoded in plant genomes (Ngou et al. 2022). Considering the large amount of PRRs and RLCKs in plant genomes, the immune signaling network could be much more sophisticated, as inferred from current findings. Therefore, much work is needed to elucidate the roles of plant RLCKs on cellular biological signaling, regarding signal convergence and divergence.

RLCKs not only participate in plant immunity but also in plant reproduction, abiotic stress responses, growth, and development. The convergent and divergent signaling mediated by RLCKs may share similarities across these processes.

## Supplementary Information

Supplementary Material 1

## Data Availability

Not applicable.

## Abbreviations

ACIK1: Avr9/Cf-9 induced kinase 1
ARCK: ABA- and osmotic-stress-inducible receptor-like cytosolic kinase
BAK1: BRI1-associated kinase 1
BIK1: Botrytis-induced kinase 1
BR: Brassinosteroid
BRI1: Brassinosteroid insensitive 1
BSK: Brassinosteroid signaling kinase
BSR1: Broad-spectrum resistance 1
CAM: Calmodulin
CC: Coiled-coil
CDG1: Constitutive differential growth 1
CERK1: Chitin elicitor receptor kinase 1
CNGC: Cyclic nucleotide-gated channel
CDPK: Calcium-dependent protein kinase
CSC: Cellulose synthesis complex
DGK5: Phosphorylate diacylglycerol kinase 5
ECD: Ectodomain
EFR: Elongation factor thermo unstable (EF-Tu) receptor
ET: Ethylene
ETI: Effector-triggered immunity
FLS2: Flagellin-sensing2
HR: Hypersensitive response
JA: Jasmonic acid
JIM2: XopJ4 immunity 2
LORE: Lipooligosaccharide-specific reduced elicitation
LYK5: Lysin motif receptor kinase5
MAPK: Mitogen-activated protein kinase
mc-3-OH-FA: Medium-chain 3-hydroxy fatty acid
NB-LRR: Nucleotide-binding leucine-rich repeat
NLR: Nucleotide-binding leucine-rich repeat receptor
OSCA: Reduced hyperosmolality-induced Ca^2+^ increase channel
PA: Phosphatidic acid
PAMP/MAMP/DAMP: Pathogen- or microbe- or damage-associated molecular pattern
PBL1: AvrPphB susceptible1 (PBS1)-like 1
PCD: Programmed cell death
PCRK: PTI compromised receptor-like cytoplasmic kinase
Pep1: Peptide1
PGN: Peptidoglycan
PRR: Pattern recognition receptor
PTI: Pattern-triggered immunity
PTI1: Pto-interacting1
Prf: Pto resistance and fenthion sensitivity
RBOHD: Respiratory burst oxidase homolog D
RIPK: RPM1-induced protein kinase
RKS1: Resistance related kinase 1
RLCK: Receptor-like cytoplasmic kinase
RLK: Receptor-like kinase
RLP: Receptor-like protein
ROS: Reactive oxygen species
SA: Salicylic acid
SAR: Systemic acquired resistance
SOBIR1: Suppressor of brassinosteroid insensitive 1
SZE1: Suppressor of ZED1-D1
TIR: Toll-interleukin-1 receptor
WT: Widetype
ZAR1: HopZ-activated resistance 1
ZED1: HopZ-ETI-deficient 1
