# Supplementary material for: Receptor-like cytoplasmic kinases mediated signaling in plant immunity: convergence and divergence
Source: Stress Biol. 2025 Jun 16;5(1):43. doi: 10.1007/s44154-025-00219-8 (PMC12170986; doi:10.1007/s44154-025-00219-8)
Supplement: Supplementary file 1 — Supplementary Material 1 [file 44154_2025_219_MOESM1_ESM.doc]

**Table legends**

**Supplementary Table 1. Summary of aspects of RLCKs**

|  | **RLCKs** | **Functions** | **Upstream RLKs** | **Stimuli** | **Downstream substrate** | **The signaling they are involved in** | **The relevant citations** |
| --- | --- | --- | --- | --- | --- | --- | --- |
| ***Arabidopsis thaliana*** | **ARCK1** | **Phosphorylated by CRK36 in response to ABA** |  | **ABA** |  | **ABA-mediated plant growth and development** | **(Tanaka et al. 2012)** |
|  | **BIK1** | **Negatively regulate BR signaling** | **BRI1** | **BRss** |  | **BR signaling** | **(Lin et al. 2013)** |
|  |  | **Induce plant immunity** | **CERK1** | **Chitin** |  | **Chitin-induced signaling** | **(Cao et al. 2014)** |
|  |  | **Phosphorylate RBOHD to promote ROS burst** | **EFR/FLS2** | **EF-Tu/flg22** | **RBOHD** | **ROS production** | **(Kadota et al. 2014)** |
|  |  | **Interact with and phosphorylated by PEPR1 to mediate ET signaling** | **PEPR1** | **Pep** |  | **Ethylene-induced immunity** | **(Liu et al. 2013)** |
|  |  | **Phosphorylate CNGC2/4 for calcium entry** |  | **Flg22** | **CNGC2/4** | **Calcium influx** | **(Tian et al. 2019)** |
|  |  | **Interact with and stabilizes CNGC20** |  | **Flg22/Pep1/AvrRpt2/AvrRpm1/AvrRps4** | **CNGC20** | **Calcium influx** | **(Yu et al. 2019; Zhao et al. 2021)** |
|  |  | **Interacts with and phosphorylate OSCA1.3** |  | **Flg22** | **OSCA1.3** | **Calcium influx** | **(Thor et al. 2020)** |
|  |  | **Regulate PA production** |  | **Flg22/AvrRpt2** | **DGK5** | **ROS production** | **(Kong et al. 2024)** |
|  |  | **Phosphorylate SHOU4L at multiple sites to inhibit its activity** |  | **Flg22/pep2/chitin/elf18** | **SHOU4L** | **Cell wall synthesis** | **(Wang et al. 2023)** |
|  |  | **BIK1-RBOHD interaction is blocked by NIS1** |  | **NIS1** |  | **ROS production** | **(Irieda et al. 2019)** |
|  |  | **Targeted by RXLR25 to inhibit the phosphorylation of BIK1** |  | **RXLR25** |  | ***Phytophthore* culture filtrate (CF)-induced immune responses** | **(Liang et al. 2021)** |
|  | **BSK1** | **Phosphorylate MAPKKK5 to regulate plant immunity** | **BRI1** | **BRs** | **MAPKKK5** | **MAPK signaling** | **(Yan et al. 2013)** |
|  | **CDG1** | **Negatively regulate PTI** | **CERK1/FLS2** | **Chitin/flg22** | **MEKK1** | **MPK4 pathway** | **(Yang et al. 2021)** |
|  |  | **Involved in AvrRpm1-induced RIN4 phosphorylation** |  | **AvrRpm1** | **RIN4** | **Effector-triggered susceptibility** | **(Yang et al. 2021)** |
|  |  | **Regulate BR signaling and plant growth** | **BRI1** | **BRs** | **BSU1** | **BR signaling** | **(Kim et al. 2011)** |
|  | **PBL1** | **Mediate MAMP-induced immunity** | **FLS2/EFR/PEPR** | **Flg22/elf18/pep1** |  | **Calcium signaling** | **(Ranf et al. 2014)** |
|  |  | **Cleaved by AvrPphB to activate RPS5** |  | **AvrPphB** |  | **Effector-triggered immunity** | **(Ade et al. 2007)** |
|  | **PBL2** | **Uridylated by AvrAC** |  | **AvrAC** | **RKS1** | **NLR-mediated signaling** | **(Wang et al. 2015)** |
|  | **PBL8/17** | **Targeted by RXLR25** |  | **RXLR25** |  | **Effector-triggered immunity** | **(Liang et al. 2021)** |
|  | **PBL13** | **Phosphorylate RBOHD to negatively regulate PTI** | **FLS2** | **Flg22** | **RBOHD** | **ROS production** | **(Lee et al. 2020)** |
|  | **PBL19** | **Link multiple PRRs to MAPK pathway** | **FLS2/CERK1/EFR/PEPR** | **Flg22/chitin/elf18/pep2** | **MAPKKK5/MEKK1** | **MAPK signaling** | **(Bi et al. 2018)** |
|  |  | **Mediate PTI** | **CERK1** | **Chitin** | **EDS1** | **EDS1-dependent plant immunity** | **(Li et al. 2020)** |
|  |  | **Upregulate *CERK1* expression to promote plant immunity** | **RLP23** | **SsNLP** | **WRKY8** | **WRKY8-dependent plant immunity** | **(Ren et al. 2024)** |
|  | **PBL27** | **Connect CERK1-LYK5 to a MAPK cascade** | **CERK1** | **Chitin** | **MAPKKK5** | **MAPK signaling** | **(Yamada et al. 2016)** |
|  |  | **Activate *PDF1.2* expression** | **HAK1** | **Polysaccharide elicitors** |  | **Ethylene-induced immunity** | **(Desaki et al. 2016)** |
|  | **PBL20** | **Interact with G proteins to maintain its stability during PTI** | **FLS2** | **Flg22** | **G proteins** | **PRR-mediated immunity** | **(Liang et al. 2016)** |
|  |  | **Associate with XopR to suppress stomatal closure** |  | **XopR** |  | **PAMP-triggered stomatal immunity** | **(Wang et al. 2023)** |
|  | **PBL30/31** | **Mediate PTI** | **SOBIR1** | **Nlp20** |  | **RLP23-mediated immunity** | **(Pruitt et al. 2021)** |
|  | **PBL34/35/36** | **Mediate PTI** | **LORE** | **mc-3-OH FAs** |  | **LORE-mediated immunity** | **(Luo et al. 2020)** |
|  | **PBS1** | **Targeted by AvrPphB to result in cleavage** |  | **AvrPphB** |  | **PTI** | **(Ade et al. 2007)** |
|  | **PCRK1/2** | **Mediate PTI** | **FLS2/EFR/PEPR** | **Flg22/elf18/pep1** |  | **PTI** | **(Sreekanta et al. 2020)** |
|  |  | **Activate SA biosynthesis** | **FLS2** | **Flg22** |  | **SA biosynthesis** | **(Kong et al. 2016)** |
|  |  | **Mediate PTI** | **RLP23** | **Nlp20** |  | **RLP23-mediated immunity** | **(Tian et al. 2021)** |
|  | **RIPK** | **Phosphorylate RBOHD to promote ROS production** | **RPM1/FLS2/EFR/CERK1/RLP23/PEPR** | **AvrRpm1/flg22/elf26/chitin/nlp20/pip/MeSA** | **RBOHD** | **Broad-spectrum RBOHD-mediated ROS signaling** | **(Li et al. 2021)** |
|  |  | **Associate with and modify an effector-targeted protein complex to initiate host immunity** | **RPM1** | **AvrB/AvrRpm1** | **RIN4** | **NLR-mediated signaling** | **(Li et al. 2011)** |
|  | **SZE1/2** | **Required for ZED1-D-activated autoimmune response and HopZ1a-triggered immunity** |  | **HopZ1a** | **ZED1/ZAR1** | **Effector-triggered immunity** | **(Liu et al. 2019)** |
|  | **ZED1** | **Act as a decay to trap HopZ1a in the ZAR1 complex** |  | **HopZ1a** |  | **Effector-triggered immunity** | **(Lewis et al. 2013)** |
| ***Oryza sativa*** | **RLCK107** | **Mediate chitin- and PGN-mediated immunity** | **CERK1** | **Chitin/PGN** |  | **Chitin- and PGN-triggered immunity** | **(Li et al. 2017)** |
|  | **RLCK118** | **Phosphorylate RbohB** | **SDS2** | ***Magnaporthe oryza*** | **RbohB** | **ROS production** | **(Fan et al. 2018)** |
|  | **RLCK176** | **Involved in PGN and chitin signaling** | **CERK1** | **Chitin/PGN** |  | **PTI** | **(Ao et al. 2014)** |
|  | **RLCK185** | **Phosphorylate CNGC9 to activate the calcium channel activity** | **CERK1** | **Chitin/flg22** | **CNGC9** | **Calcium influx** | **(Wang et al. 2019)** |
|  |  | **Phosphorylate OsMAPKKKε** | **CERK1** | **Chitin/flg22** | **MAPKKKε** | **Chitin-induced MAPK activation** | **(Wang C et al. 2017)** |
|  |  | **Targeted by Xoo1488 to inhibit RLCK185 phosphorylation by CERK1** | **CERK1** | **Chitin/Xoo1488** |  | **Chitin- and peptidoglycan-induced immunity** | **(Yamaguchi et al. 2013)** |
|  | **RLCK278 (BSR1)** | **Positively regulate rice innate immunity** | **CERK1** | **Chitin/rice blast fungus** |  | **ROS production; expression of defense-related genes** | **(Kanda et al. 2017)** |
| ***Solanum lycoperscium*** | **ACIK1** | **Positively regulate Cf9/Cf4-mediated resistance** |  | **Avr9/Avr4** |  | **Cf9/Cf4-mediated resistance** | **(Rowland et al. 2005)** |
|  | **JIM2** | **Function with ZAR1 to recognize the effector XopJ4** |  | **XopJ4** |  | **Effector-triggered immunity** | **(Schultink et al. 2019)** |
|  | **Mai1** | **Function with CC-NLRs to regulate tomato PCD** |  | **AvrPto/AvrPtoB** | **MAPKKKα** | **NLR mediated resistance** | **(Roberts et al. 2019)** |
|  | **PTI1** | **Regulate PTI signaling** |  | **Flg22/flgⅡ-28** |  | **ROS production** | **(Schwizer et al. 2017)** |
|  | **Pto** | **Targeted by the effector AvrPto/AvrPtoB** |  | **AvrPto/AvrPtoB** |  | **Effector-triggered immunity** | **(Kim et al. 2002; Scofield et al. 1996; Tang et al. 1996)** |
| ***Triticum aestivum*** | **IPK1** | **Enhance the kinase activity of PsIPK1 for CBF1d phosphorylation** |  | **Spg1** | **CBF1d** | **Effector-triggered susceptibility** | **(Wang et al. 2022)** |
| ***Nicotiana benthamiana*** | **RLCK-VII-6, -7, and -8** | **Regulate flg22- and chitin-induced ROS production** | **SOBIR1** | **Flg22/chitin** |  | **ROS production** | **(Huang et al. 2024)** |
| ***Marchantia polymorpha*** | **PBLa** | **Phosphorylate RBOH1 at conserved residues** |  | **Chitin** | **RBOH1** | **ROS production** | **(Chu et al. 2023)** |
